# The Truncated Peptide AtPEP1^(9–23)^ Has the Same Function as AtPEP1^(1–23)^ in Inhibiting Primary Root Growth and Triggering of ROS Burst

**DOI:** 10.3390/antiox13050549

**Published:** 2024-04-29

**Authors:** Junmei Cui, Ermei Sa, Jiaping Wei, Yan Fang, Guoqiang Zheng, Ying Wang, Xiaoxia Wang, Yongjie Gong, Zefeng Wu, Panfeng Yao, Zigang Liu

**Affiliations:** 1 State Key Laboratory of Aridland Crop Science, Gansu Agricultural University, Lanzhou 730070, China; cuijm@gsau.edu.cn (J.C.); sem@st.gsau.edu.cn (E.S.); weijp@gsau.edu.cn (J.W.); fangy@gsau.edu.cn (Y.F.); zhenggq@st.gsau.edu.cn (G.Z.); wangying2@st.gsau.edu.cn (Y.W.); wxx@st.gsau.edu.cn (X.W.); gongyj@st.gsau.edu.cn (Y.G.); wuzf@gsau.edu.cn (Z.W.); yaopf@gsau.edu.cn (P.Y.); 2 College of Agronomy, Gansu Agricultural University, Lanzhou 730070, China

**Keywords:** plant elicitor peptide 1, primary root, root tip, reactive oxygen species burst

## Abstract

Currently, the widely used active form of plant elicitor peptide 1 (PEP1) from *Arabidopsis thaliana* is composed of 23 amino acids, hereafter AtPEP1^(1–23)^, serving as an immune elicitor. The relatively less conserved N-terminal region in AtPEP family indicates that the amino acids in this region may be unrelated to the function and activity of AtPEP peptides. Consequently, we conducted an investigation to determine the necessity of the nonconserved amino acids in AtPEP1^(1–23)^ peptide for its functional properties. By assessing the primary root growth and the burst of reactive oxygen species (ROS), we discovered that the first eight N-terminal amino acids of AtPEP1^(1–23)^ are not crucial for its functionality, whereas the conserved C-terminal aspartic acid plays a significant role in its functionality. In this study, we identified a truncated peptide, AtPEP1^(9–23)^, which exhibits comparable activity to AtPEP1^(1–23)^ in inhibiting primary root growth and inducing ROS burst. Additionally, the truncated peptide AtPEP1^(13–23)^ shows similar ability to induce ROS burst as AtPEP1^(1–23)^, but its inhibitory effect on primary roots is significantly reduced. These findings are significant as they provide a novel approach to explore and understand the functionality of the AtPEP1^(1–23)^ peptide. Moreover, exogenous application of AtPEP1^(13–23)^ may enhance plant resistance to pathogens without affecting their growth and development. Therefore, AtPEP1^(13–23)^ holds promise for development as a potentially applicable biopesticides.

## 1. Introduction

In 2006, Huffaker et al. first isolated and characterized an endogenous peptide, named plant elicitor peptide 1 (PEP1), in *Arabidopsis thaliana* [1]. This peptide, consisting of 23 amino acids, hereafter is named AtPEP1^(1–23)^. Approximately 20 years later, Hander et al. demonstrated that the precursor gene undergoes cleavage by METACASPASE4 (MC4) at a conserved arginine (R69) to produce processed AtPEP1^(1–23)^. Destruction of the catalytic cysteine residue in MC4 or the conserved arginine residue preceding the AtPEP1^(1–23)^ sequence blocks the cleavage process of precursor of peptide 1 (AtPROPEP1) [2,3]. Furthermore, Shen and colleagues demonstrated that in addition to MC4, the coexpression of AtPROPEP1 with other type-II MCs, including MC5 to MC9, significantly enhances the generation of processed AtPEP1^(1–23)^ [3]. Pathogen- or damage-associated molecular pattern treatments have been shown to increase the expression of AtPROPEP1 and its receptors, such as flg22 and PIP7 [3,4]. Moreover, flg22 enhances the MC4-mediated processing of AtPROPEP1 [3]. Amplifying immune responses is a common employed strategy by animals and plants upon detecting a limited number of invading pathogens [5].

The recognition of APEP1^(1–23)^ relies on the leucine-rich repeat receptor-like kinase (LRR-RLK) PEPR (PEP Receptor) 1/2 [6,7], which triggers convergent immune signaling events as other elicitors, including changes in cytoplasmic Ca^2+^ levels, activation of mitogen-activated protein kinases (MAPK) cascades, induction of defense-related genes, production of reactive oxygen species (ROS) and nitric oxide, enhancement of cell wall with callose deposition, and stomatal closure to prevent pathogen invasion [8,9,10,11]. AtPEP1^(1–23)^ not only activates immune responses in leaves but also induces immune responses in roots [12]. Furthermore, the application of APEP1^(1–23)^ in roots can also induce systemic immune signaling in shoots, enhancing resistance against the bacterial pathogen *Pst DC3000* [12,13]. Interestingly, AtPEPR2 is predominantly responsible for perceiving APEP1^(1–23)^ in the roots, unlike in the leaves [12]. The immune responses activated by AtPEP1^(1–23)^ confer resistance against bacterial, fungal, and oomycete, as well as herbivores [1,7]. Arabidopsis mutants with single mutants in AtPEPR1 and AtPEPR2, as well as their double mutants, are more sensitive to the fungus *Phymatotrichopsis omnivora* compared to the wild-type plants. The mutants showed a 5-day earlier death than the wild type, indicating that the PEP-mediated immune response confers resistance against *P. omnivora* [14]. Pretreatment of maize plants with ZmPEP1 enhances their ability to resist southern leaf blight caused by *Cochliobolis heterostrophus* and anthracnose stalk rot caused by *Colletotrichum graminicola* [15]. When grown in soil, Arabidopsis plants with constitutive overexpression of either AtPROPEP1 or AtPROPEP2 exhibited an enhancement in both root and aerial growth compared to wild-type plants when subjected to inoculation with the oomycete root pathogen *Pythium irregulare* [1]. This suggests that AtPEP1^(1–23)^ signaling plays a crucial role in conferring resistance to *P. irregulare*. It is AtPEPR2, not AtPEPR1, that recognizes the symptom determinant C4 in geminiviruses and enhances its internalization, thereby suppressing the infection of Beet severe curly top virus [16]. Pretreatment of potato roots with StPEP1 reduces the infection of the root-knot nematode *Meloidogyne chitwoodi* [17]. The immune signal transduction mediated by AtPEP1^(1–23)^-AtPEPRs still requires the involvement of other kinases, such as BAK1 (BRI1-associated receptor kinase), PCRK1 (pattern-triggered immunity (PTI) compromised receptor-like cytoplasmic kinase 1), BSK5 (brassinosteroid-signaling kinase 5), and BIK1 (botrytis-induced kinase 1) [18,19,20,21]. Null mutations in these genes result in weakened or abolished immune responses triggered by AtPEP1^(1–23)^. In summary, the intricate immune signaling pathway involving PEP and PEPRs plays a significant role in enhancing plant resistance against pathogens.

In addition to immune responses, the AtPEP1^(1–23)^-AtPEPRs pathway inhibits root growth, including the shortening of primary roots, induction root hair formation, and reduction in lateral root numbers [22,23]. This may be due to the high expression of AtPEP1^(1–23)^ receptors in the root vascular system, while the receptors of the immune elicitors flg22 and elf18 are not [24]. The application of exogenous AtPEP1^(1–23)^ significantly affects the division of stele cells, resulting in a decrease in their number [24]. Further research results indicate that specific types of root cells expressing AtPEPR2 seem to have the ability to perceive AtPEP1^(1–23)^. Exogenous application of AtPEP1^(1–23)^ stimulates root hair formation, inhibits primary root growth, and triggers immune responses in all cell types expressing AtPEPR2, except for root hair cells which only undergo immune responses [25]. In addition to AtPEPR2, the formation of root hairs mediated by AtPEP1^(1–23)^ also requires the involvement of kinases BAK1 and BIK1, as well as ethylene (ET), auxin, and regulators of root hair differentiation [25]. A variety of hormones, including auxin, ET, brassinosteroid (BR), and cytokinin (CK), coordinate to determine the fate and dynamics of developmental cell during root growth in response to environmental stimuli [26,27,28,29]. Recent research conducted on Arabidopsis seedlings has showed that AtPEP1^(1–23)^ inhibits root growth by interacting with PIN-dependent local distribution of auxin [30,31]. The accumulation of auxin induced by AtPEP1^(1–23)^ in the transition zone (TZ) depends on apoplastic acidification [31]. Therefore, the interplay between auxin and pH signaling plays a crucial role in regulating the inhibition of root growth mediated by AtPEP1^(1–23)^. The inhibitory effect on primary root growth caused by AtPEP1^(1–23)^ is negated in seedlings harboring mutations in two major genes responsible for encoding ROS-generating enzymes (RBOHD and RBOHF), as well as BIK1 [12]. Additionally, BIK1 physically interacts with AtPEPRs and RBOHD/F [12]. Hence, the cross-talk between AtPEP1^(1–23)^ signaling and ROS signaling contributes to the regulation of primary root growth. 

The Arabidopsis genome contains eight AtPEP precursor genes (AtPROPEP1-8), and the synthetic AtPEP peptides have similar inducing effects on pattern-triggered immune responses [32]. The synthesized AtPEP peptides have a conserved motif, SSG(x)_2_G(x)_2_N, at the C-terminal, but the conservation at the N-terminal is very low [32]. We speculate that the low conserved N-terminus region of AtPEPs may not be essential for their function. Compared to theoretically processed peptide, AtPEP7, which lacks two amino acids at the N-terminus, it still exhibits high activity [32]. This result supports our speculation. Site-directed mutagenesis and stepwise truncation can be used to study the impact of amino acids on peptide function or activity [33]. Truncation refers to the removal of one or more amino acids from the peptide, followed by evaluation of the functional or activity changes in the truncated peptide. This approach helps us understand the relationship between peptide structure and function, and elucidates the roles of different amino acids in peptide function. Therefore, in this study, we will adopt the strategy of truncating the peptide to investigate the contribution of each amino acid in the activity of AtPEP1^(1–23)^, a peptide consisting of 23 amino acids that is widely used. Through a series of experiments involving progressive truncation, we will compare the differences in ROS burst and primary root growth inhibition between the truncated peptides and the original peptide. Our research aims to uncover the contribution of each amino acid in the peptide AtPEP1^(1–23)^ to its function, in order to identify the shortest peptide sequence that retains almost all of the original peptide activity. This is of great significance for further study of the recognition and regulatory mechanisms of AtPEP1^(1–23)^. Additionally, we also aim to obtain truncated peptides that only retain immune response activation activity without or with significantly reduced developmental inhibition activity. This will provide guidance for the development of pesticides.

## 2. Materials and Methods

### 2.1. Materials and Treatments

Peptide elicitors were ordered from SYNBIO TECHNOLOGIES (Suzhou, China) with a purity of 95%. The peptides were prepared as a stock solution with a concentration of 1 mM.

All plants used in this study are of the Arabidopsis thaliana accession Columbia-0 (Col-0) background, including the wild-type, mutant line *pepr1-2pepr2-2* (*pepr1/2*, AT1G73080 and AT1G17750) [6] and transgenic lines expressing *WOX5*::GFP (AT3G11260) [34] and *DR5*::GFP [35]. DR5 was created by petforming site-directed mutations in a natural composite auxin response element (AuxRE) found in the soybean GH3 promoter. DR5 consisted of tandem direct repeats of 11 bp (CCTTTTGTCTC)

For sterile seedlings, the seeds were first surface-sterilized with 75% ethanol twice. Then, after cold treatment at 4 °C for 2 days, the seeds were germinated on 1/2 Murashige-Skoog (MS) medium supplemented with 0.8% agar and 1% sucrose. For four-week-old plants, the seeds after cold treatment at 4 °C for 2 days were grown in a 1:1 mixture of sand: humus. Both growth environments were carried out in a growth cabinet with 12 h of light, a temperature of 22 °C, and 60% humidity. 

### 2.2. Measurement of Root Length and Microscopic Observation of Root Tips

Five-day-old seedlings were transferred from 1/2 MS medium to 1/2 MS medium supplemented with or without elicitor for four days. The transferred seedlings were photographed every day for a total of four days. The primary root length was measured using ImageJ software (V 1.8.0). After four days of elicitor treatment, the root tips of seedlings were observed by microscope and the fluorescence from root tip cells of GFP tag seedlings were excited with a 488 nm laser. Emission was detected between 495 and 550 nm. The images were acquired with an OLYMPUS FV10-MCPUS confocal laser scanning microscope. Images were processed using LSM image examiner software (FV10-ASW3.1) and Adobe Photoshop CS4 version 11.0.2 (Adobe Systems, San Jose, CA, USA). 

### 2.3. ROS Burst Measurements

The measurement of ROS burst was carried out using a luminol dependent assay [33]. Briefly, leaf samples were cut into small pieces and placed in a 96-well plate containing 0.1 mL reaction solution (2 µg/mL horseradish peroxidase, 200 µM luminol L-012). Luminescence signal was immediately measured upon the addition elicitor peptides and recorded for a period of 30 min. The total relative light units (RLUs) measured within a 15 min time was plotted.

### 2.4. Sequence Alignment

The protein sequences of AtPROPEP family were downloaded from TAIR (https://www.arabidopsis.org/, accessed on 10 May 2022). Then, we predicted the processed peptides based on the processed AtPEP1^(1–23)^ peptide [2]. Sequence alignment was performed using ClustalW version 2.0.10. 

### 2.5. Molecular Docking

The structures of target proteins were constructed using AlphaFold2. The AMBER10 force field [36] was used for deprotonation, handling missing atoms, completing missing groups, and energy minimization, with the default values of the Molecular Operating Environment (MOE) version 2019.01 (http://www.chemcomp.com, accessed on 1 September 2023). A topology file was exported as a parmtop file.

A total of 100 putative binding modes were generated using the HDOCK server (http://hdock.phys.hust.edu.cn/, accessed on 6 December 2023), with the proteins set to be rigid and the docking contact point set to be the whole surface [37]. The binding modes were scored and ranked by ITScorePP, a proprietary statistical knowledge-based protein–protein scoring function [38]. The binding modes with the lowest energy were selected for optimization using the Minimization module in MOE 2019.01 to resolve the potential unreasonable contact in the spatial structure resulting from rigid docking. Amber10:ETH was used as the force field for energy minimization, and water molecules were used as the solvation model. The optimization was performed using steepest descent and conjugate gradient methods, with a maximum number of 5000 iterations. Finally, the optimization results were visualized using Pymol2.1 software.

### 2.6. Protein Domain Analysis

DeepTMHMM (https://dtu.biolib.com/DeepTMHMM, accessed on 13 December 2023) is a commonly used tool for predicting the structure of transmembrane proteins [39]. It can analyze protein sequences and predict the locations of transmembrane regions and transmembrane α-helices within the protein. This is crucial for understanding the structure and function of receptor proteins. AtPEPR1 and AtPEPR2 are receptors for AtPEP peptides and belong to the typical LRR-RLKs. To analyze their structures, we can utilize the DeepTMHMM.

### 2.7. Statistical Analysis

All experiments were conducted in triplicate without special instructions. One-way or two-way ANOVA analyses were performed using GraphPad Prism 9 to determine the statistical significances. *, **, ***, and **** indicate the significant differences as *p* < 0.05, *p* < 0.01, *p* < 0.001, and *p* < 0.0001 levels, respectively.

## 3. Results

### 3.1. Sequence Alignment of Eight AtPEP Peptides

**Figure 1:**
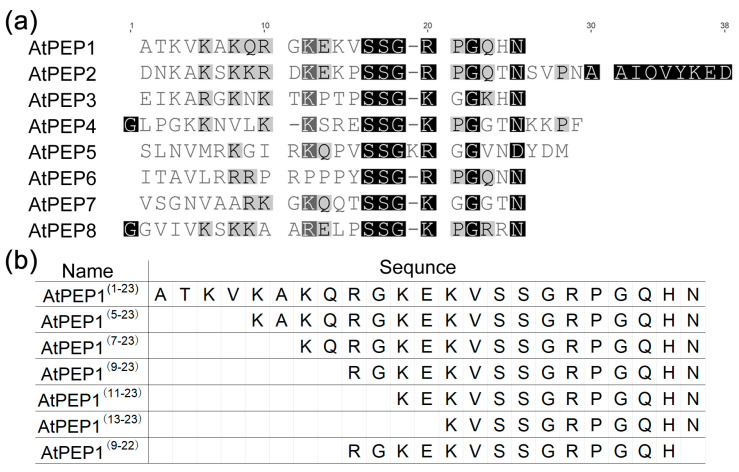
Sequence information of AtPEP family peptides. (**a**) Alignment of AtPEP family peptides, the darker the color, the higher the similarity; (**b**) Peptide sequence information used in this study.

The genome of *A. thaliana* contains eight AtPROPEP proteins, and the sequences of these eight precursor proteins were downloaded from TAIR. According to the results of Bartels et al. and Hander et al. [2,32], theoretically processed PEP peptide sequences were used for sequence alignment analysis. The C-terminal of eight AtPEP peptides was highly conserved (SSG(x)_2_G(x)_2_N), while the N-terminal showed very low similarity (Figure 1a). Because these peptides all rely to some degree on the receptors AtPEPR1 and AtPEPR2 to exert their function [32], we speculate that some amino acids at the N-terminal of AtPEPs may not be essential for their functions. To verify this hypothesis, we took AtPEP1^(1–23)^ as an example to study the function by truncation. The peptide sequences are shown in Figure 1b. 

### 3.2. The Inhibitory Effect of AtPEP1^(9–23)^ on Primary Root Growth Was Similar to AtPEP1^(1–23)^

**Figure 2:**
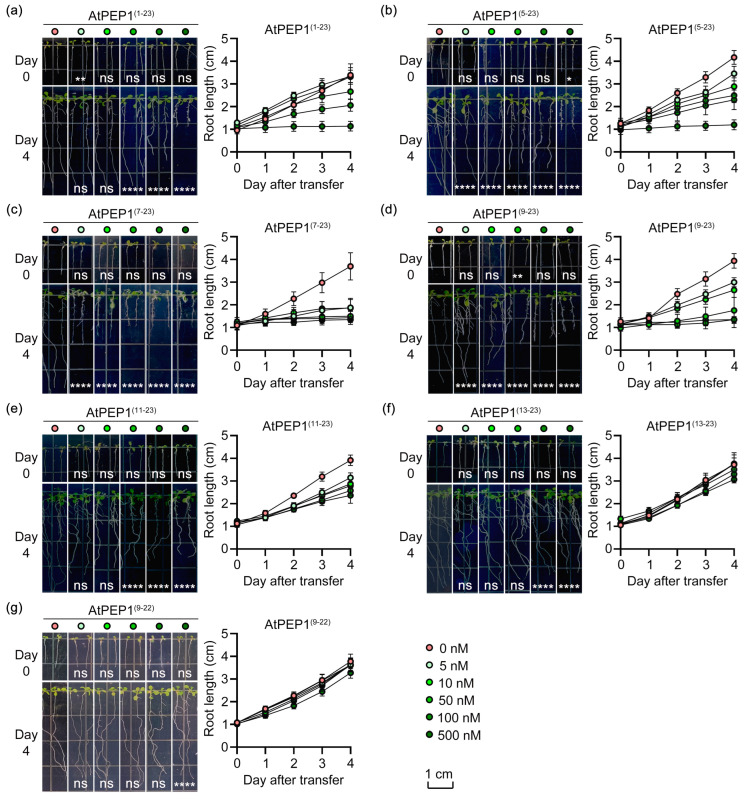
Phenotypes and data of primary roots after treatment with AtPEP1^(1–23)^ (**a**), AtPEP1^(5–23)^ (**b**), AtPEP1^(7–23)^ (**c**), AtPEP1^(9–23)^ (**d**), AtPEP1^(11–23)^ (**e**), AtPEP1^(13–23)^ (**f**), and AtPEP1^(9–22)^ (**g**). Five-day-old seedlings were transferred from 1/2 MS to culture media with different concentrations of peptides. The left side shows the phenotypes of the primary roots, while the right side presents the data on primary root length. Results are presented as mean ± standard deviation (SD). “ns” represents no significant difference compared to the control treatment (0 nM). *, **, **** indicate significant differences at *p* < 0.05, *p* < 0.01, and *p* < 0.0001 levels, respectively, compared to the control treatment (two-way ANOVA, n = 20).

Our previous research results have shown that AtPEP1^(1–23)^ can inhibit primary root growth. Therefore, we presented representative seedlings transferred to the medium with or without peptide on day 0 and day 4, and measured their growth rate (Figure 2). There was almost no difference in initial root length under different concentrations (Table S1). Over time, AtPEP1^(1–23)^ at 50 nM could inhibit primary root growth, and the inhibition effect became more pronounced with the increasing concentration. When the concentration reached 500 nM, it completely inhibited the primary root growth (Figure 2a). The results of AtPEP1^(5–23)^, AtPEP1^(7–23)^, and AtPEP1^(9–23)^ were similar to AtPEP1^(1–23)^, but their efficiencies were higher, especially AtPEP1^(7–23)^, which inhibited root length by approximately 49% after 4 days at a concentration of 5 nM (Figure 2b–d). AtPEP1^(11–23)^ could also inhibit the growth of primary root at 5 nM, but its inhibitory effect did not change significantly with increasing concentration (Figure 2e). Generally speaking, the inhibitory effect of AtPEP1^(11–23)^ on primary root growth was lower than that of AtPEP1^(1–23)^. Unlike other peptides, AtPEP1^(13–23)^ did not show an obvious inhibitory effect on primary root growth, even at concentrations as high as 500 nM (Figure 2f). 

In order to investigate the impact of conserved C-terminal amino acids on the growth of the primary root, we conducted a truncation experiment based on AtPEP1^(9–23)^, resulting in the creation of a truncated peptide known as AtPEP1^(9–22)^. The experimental findings demonstrated that AtPEP1^(9–22)^ exhibited a similar inhibitory effect on primary root growth compared to AtPEP1^(13–23)^, implying that the conserved C-terminal amino acid plays a crucial role in the inhibition of primary root growth by AtPEP1^(1–23)^. 

In addition, we found that, in addition to AtPEP1^(13–23)^ and AtPEP1^(9–22)^, the other peptides also exhibited significant inhibitory effects on the shoot (Figure S1, Table S7). The above results indicate that the first eight N-terminal amino acids of AtPEP1^(1–23)^ do not significantly influence primary root growth, while the C-terminal conserved amino acid does.

### 3.3. AtPEP1^(9–23)^ Promoting Premature Root Tip Cells

**Figure 3:**
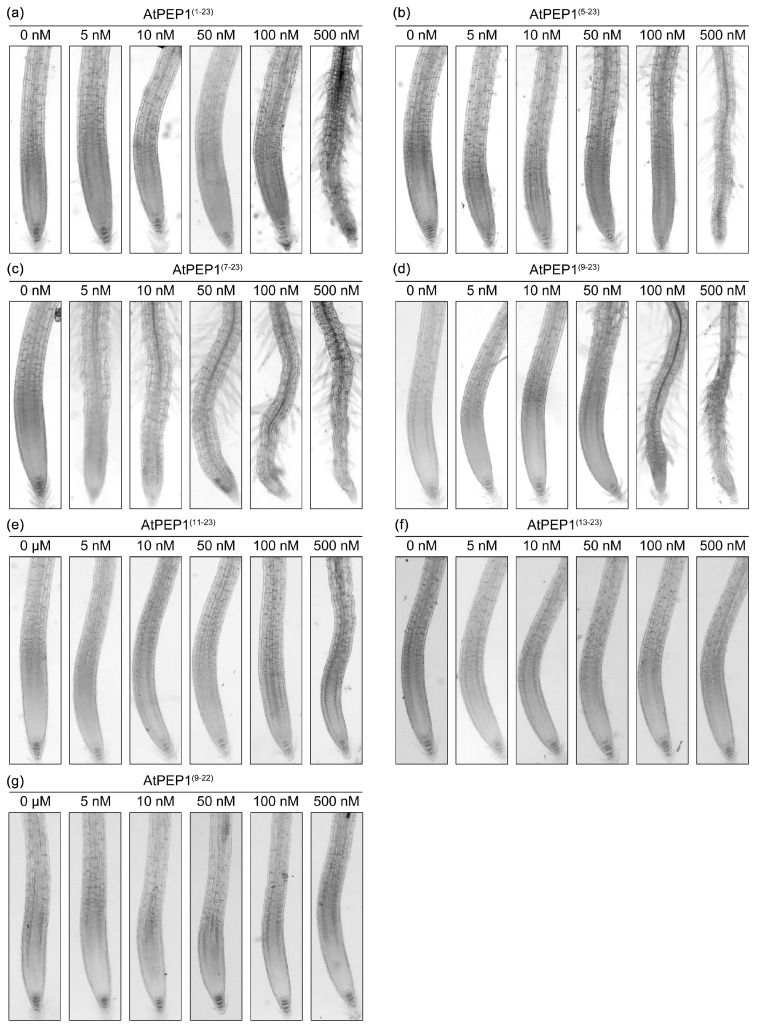
Root tip phenotypes. Five-day-old seedlings were treated with peptide AtPEP1^(1–23)^ (**a**), AtPEP1^(5–23)^ (**b**), AtPEP1^(7–23)^ (**c**), AtPEP1^(9–23)^ (**d**), AtPEP1^(11–23)^ (**e**), AtPEP1^(13–23)^ (**f**), and AtPEP1^(9–22)^ (**g**) for 4 days, followed by observation of root tip phenotypes (n = 20). The magnification of the eyepiece and objective lenses of a microscope is both 10 times.

To analyze the mechanism of the above peptides inhibiting primary root growth, we first observed the morphology of root tip (Figure 3). It can be observed from Figure 3a that high concentration of AtPEP1^(1–23)^ promoted the generation of root hairs. The region with root hairs represents a mature area, indicating that AtPEP1^(1–23)^ promotes the maturation of root tip cells in a concentration-dependent manner. Peptides AtPEP1^(5–23)^, AtPEP1^(7–23)^, and AtPEP1^(9–23)^ were more efficient than AtPEP1^(1–23)^ in promoting the maturation of root tip cells (Figure 3b–d). However, AtPEP1^(11–23)^, AtPEP1^(13–23)^, and AtPEP1^(9–22)^ did not promote the generation of root hairs, even at a high concentration of 500 nM (Figure 3e–g). The results of root tip morphology were consistent with the results of primary root length (Figure 2). 

### 3.4. AtPEP1^(9–23)^ Regulating Auxin and Quiescent Centre in Root Tips

**Figure 4:**
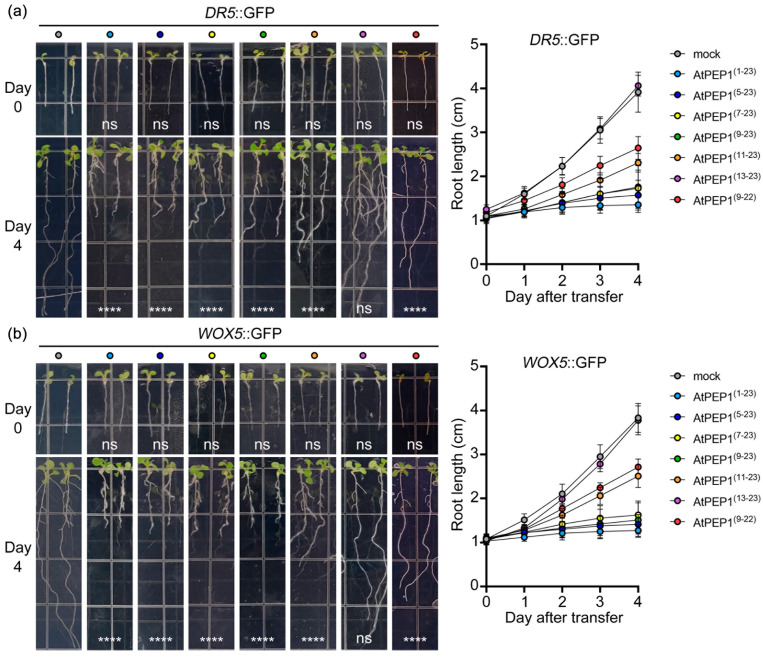
Root phenotypes and root length of transgenic lines *DR5*::GFP (**a**) and *WOX5*::GFP (**b**). The left panel shows the primary root phenotypes on day 0 and day 4 after transferring 5-day-old seedlings to medium containing 500 nM peptide. The right panel presents the primary root length data after transfer. Results are presented as mean ± SD. “ns” represents no significant difference compared to the control treatment (mock); **** indicates significant differences at *p* < 0.0001 level compared to the control treatment (two-way ANOVA, n = 20).

**Figure 5:**
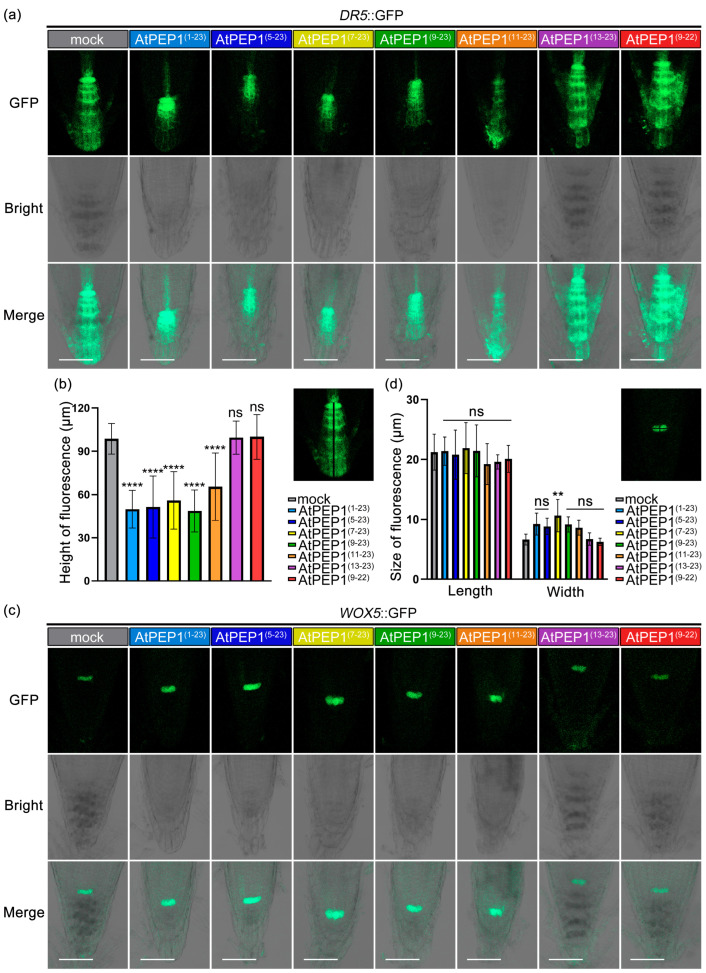
The GFP expression in root tips of transgenic lines *DR5*::GFP and *WOX5*::GFP. Five-day-old seedlings were treated with 500 nM peptide for four days, followed by observation of *DR5*::GFP (**a**) and *WOX5*::GFP (**c**) using confocal microscopy. The scale was 50 μm. (**b**,**d**) The GFP fluorescence regions were measured as indicated, and the results are presented as mean ± SD. “ns” represents no significant difference compared to the control treatment (mock); **, **** indicate significant differences compared to the control treatment (n = 20), with *p* < 0.01 and *p* < 0.0001, respectively. One-way ANOVA was conducted for (**b**), and two-way ANOVA for (**d**).

Since the initiation of root growth is strongly influenced by the accumulation of auxin in the cells, experiments were conducted to investigate whether peptide treatment could disrupt the formation of an auxin maxima in root tips. Five-day-old *DR5*::GFP lines (which serve as reporters for auxin maxima in root tips) in Col-0 background were cultured on 1/2 MS medium supplemented with 500 nM peptide. The root length phenotype of the *DR5*::GFP lines, after peptide treatments, resembled that of wild-type Col-0 (Figure 2 and Figure 4a, Table S2). Compared with the control group, the maximum levels of auxin in root tips were significantly reduced in the treatments of AtPEP1^(1–23)^, AtPEP1^(5–23)^, AtPEP1^(7–23)^, AtPEP1^(9–23)^, and AtPEP1^(11–23)^ (Figure 5a,b). However, AtPEP1^(13–23)^ and AtPEP1^(9–22)^ did not show significant changes (Table S3). These results were obtained by detecting the reporter gene *DR5*::GFP.

The observed morphological changes in *WOX5*::GFP root tips prompted us to examine the activity of the root meristematic quiescent center (QC) [35]. Similar to the findings in the *DR5*::GFP lines, the phenotype and length of primary root in *WOX5*::GFP lines did not show any significant difference when subjected to peptide treatment, as compared to the wild-type Col-0 (Figure 2 and Figure 4b). The expression of *WOX5*::GFP was detected in the two stem cells of the QC, and treatment with peptides AtPEP1^(1–23)^, AtPEP1^(5–23)^, AtPEP1^(7–23)^, AtPEP1^(9–23)^, and AtPEP1^(11–23)^ led to an increase in *WOX5* expression, manifested by an increase in signaling width (Figure 5c,d). These results suggest that treatment with AtPEP1^(1–23)^, AtPEP1^(5–23)^, AtPEP1^(7–23)^, AtPEP1^(9–23)^, and AtPEP1^(11–23)^ may affect the activity of quiescent center cells.

### 3.5. The Supposed Binding Affinity between AtPEP1^(13–23)^ and the Receptors May Be Decreased

**Figure 6:**
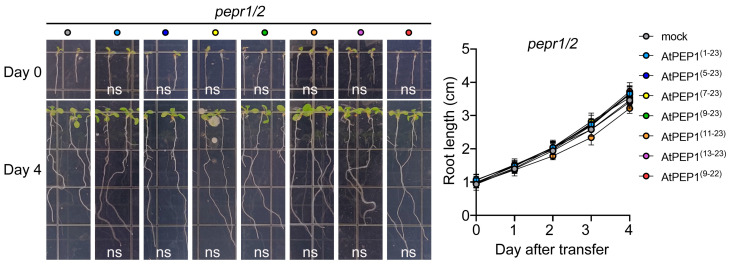
Phenotype and root length of double mutant *pepr1/2* primary roots. On the left side, the primary root phenotypes were observed on Day 0 and Day 4 after transferring 5-day-old seedlings to culture media containing 500 nM peptide. On the right side, the data for primary root length were recorded for the seedlings transferred to culture media containing 500 nM peptide. Results are expressed as mean ± SD; “ns” represents no significant difference compared to the mock treatment (two-way ANOVA, n = 20).

**Figure 7:**
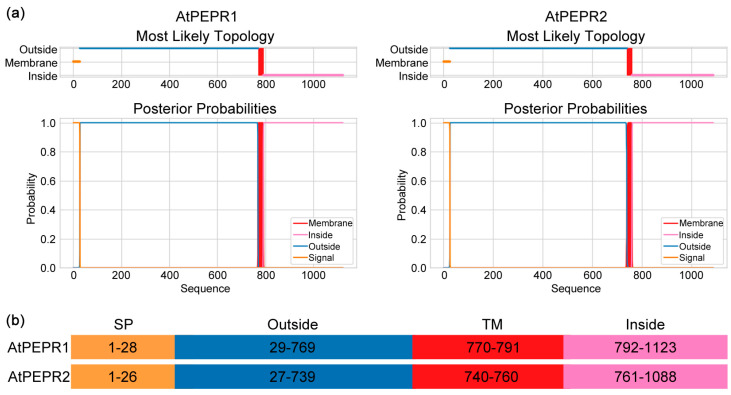
TMHMM analysis of AtPEPR1 and AtPEPR2. (**a**) Topology model and probability of the target proteins; (**b**) distribution of amino acids in signal peptide (SP), extracellular domain (outside), transmembrane domain (TM), and intracellular domain (inside).

From Figure 2, Figure 3 and Figure 4, it can be seen that truncating the N-terminal eight amino acids of AtPEP1^(1–23)^ does not affect its ability to inhibit primary root growth. However, further truncation of the N-terminal weakens or even abolishes its inhibitory effect on primary root growth. Since the functionality of AtPEP1^(1–23)^ relies on the receptors AtPEPR1 and AtPEPR2, we speculate that the binding affinity between truncated peptides and the receptors may be decreased, which could be one of the reasons for the aforementioned results. Therefore, we first investigated the inhibitory effects of peptides on primary root growth in the *pepr1/2* double mutant. From the phenotype and data of the primary roots, there was no significant difference between the peptide treatments and the control treatment (Figure 6, Table S4), indicating that truncated peptides also rely on AtPEPR1 and AtPEPR2 to inhibit primary root growth. Based on previous studies, it is known that the extracellular domains of receptors are responsible for ligands’ recognition and binding. Hence, the structures of AtPEPR1 and AtPEPR2 were analyzed using TMHMM. The results showed that both proteins contain extracellular domains, transmembrane domains, and intracellular domains with high confidence (Figure 7a). The amino acids from 29 to 769 in AtPEPR1 and from 27 to 729 in AtPEPR2 are likely to interact with ligands (Figure 7b).

**Figure 8:**
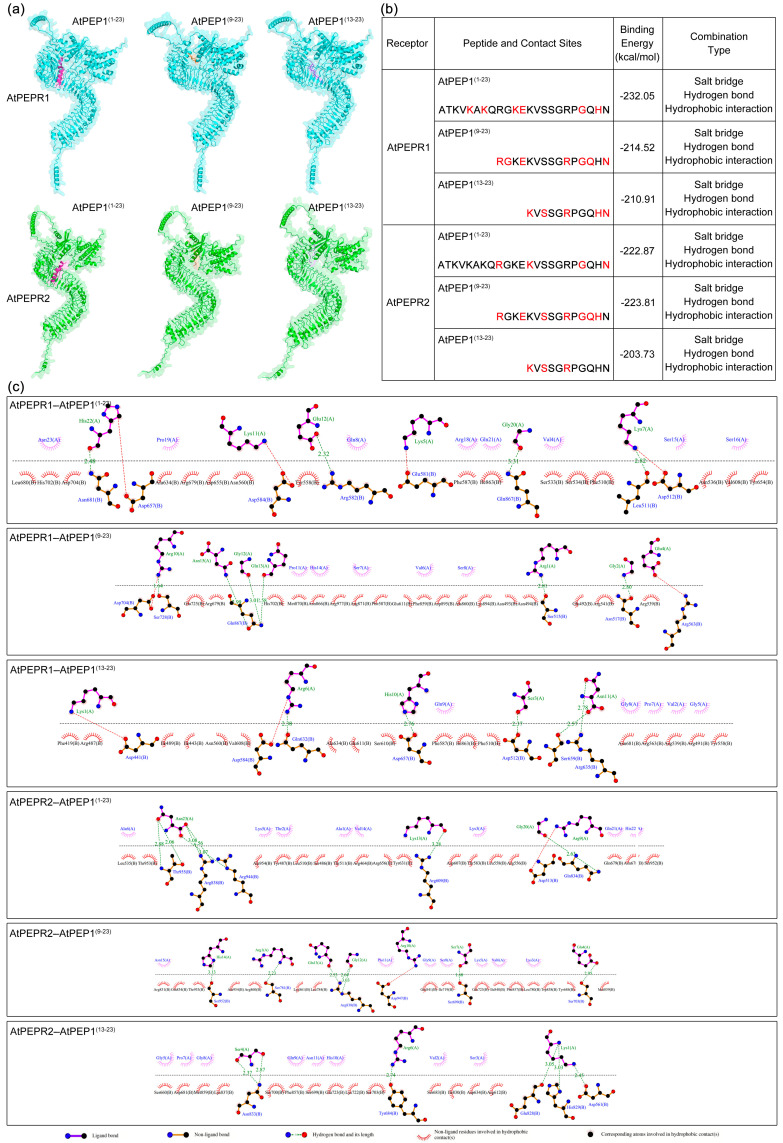
Molecular docking of ligand peptides with receptor proteins. (**a**) Optimal docking poses between ligand peptides and receptor proteins. Receptor proteins AtPEPR1 and AtPEPR2 are represented by blue and green, respectively. Ligand peptides AtPEP1^(1–23)^, AtPEP1^(9–23)^, and AtPEP1^(13–23)^ are represented by pink, orange, and purple, respectively. (**b**) Complexes in terms of contact sites, energy, and binding properties. The possible contact sites of peptides are highlighted in red. (**c**) Binding interactions between peptide ligands and AtPEPR1 and AtPEPR2. The blue ball, nitrogen atom; red ball, oxygen atom; black ball, carbon atom. The dotted black line above represents the peptide ligand, while the line below represents the receptor.

In this study, we explored the potential interactions between peptides AtPEP1^(1–23)^, AtPEP1^(9–23)^ (which displayed comparable inhibitory activity on root growth as AtPEP1^(1–23)^), and AtPEP1^(13–23)^ (which exhibited noticeably diminished inhibitory activity on root growth) and receptors through molecular docking simulations (Figure 8). The results showed that the peptides AtPEP1^(1–23)^, AtPEP1^(9–23)^, and AtPEP1^(13–23)^ were capable of binding to receptors AtPEPR1 and AtPEPR2 (Figure 8a). Possible binding sites between peptides and receptors were highlighted in red, with binding energies varying from −232.05 to −203.73 kcal/mol (Figure 8b). The molecular structures revealed that the peptides might interact with receptors through hydrogen bonding, salt bridges, and hydrophobic interactions (Figure 8c). The amino acid residues Lys5, Lys11, Glu12, and Gly20 of AtPEP^(1–23)^ formed hydrogen bonds with the residues Glu81, Asp584, Arg582, and Gln867 of AtPEPR1, respectively. The Lys7 residue of AtPEP^(1–23)^ formed hydrogen bonds with the residues Leu511 and Asp512 of AtPEPR1. The His22 residue of AtPEP^(1–23)^ peptide formed hydrogen bonds with the residues Asn681 and Asp657 of AtPEPR1. The residues Arg1, Gly2, and Glu4 of AtPEP^(9–23)^ established hydrogen bonds with Ser515, Asn517, and Arg563 of AtPEPR1, respectively. The residue Arg10 of AtPEP^(9–23)^ formed hydrogen bonds with Ser728 and Asp704 of AtPEPR1. The residues Gly12, Gln13, and Asn15 of AtPEP^(9–23)^ participated in hydrogen bonding interactions with Gln867 of AtPEPR1. The amino acid residues Lys1, Ser3, and Gln9 of the peptide AtPEP^(13–23)^ formed hydrogen bonds with the residues Asp441, Asp512, and Asp657 of AtPEPR1, respectively. The residue Arg6 of AtPEP^(13–23)^ formed hydrogen bonds with the residues Asp584 and Gln632 of AtPEPR1. Moreover, the residue Asn11 of AtPEP^(13–23)^ participated in hydrogen bonding interactions with the residues Ser659 and Arg635 of AtPEPR1. According to the molecular docking results, it can be observed that there were changes in the binding sites between the peptides AtPEP^(1–23)^, AtPEP^(9–23)^, AtPEP^(13–23)^, and the receptor AtPEPR1. Moreover, the binding sites between AtPEP^(13–23)^ and AtPEPR1 decreased. This could be one of the reasons for the decreased inhibitory effect of AtPEP^(13–23)^ on primary root growth. The molecular docking results of the peptides AtPEP^(1–23)^, AtPEP^(9–23)^, and AtPEP^(13–23)^ with the receptor AtPEPR2 were similar to those with AtPEPR1.

### 3.6. The Truncated Peptides Caused ROS Burst Pattern Similar to AtPEP1^(1–23)^

**Figure 9:**
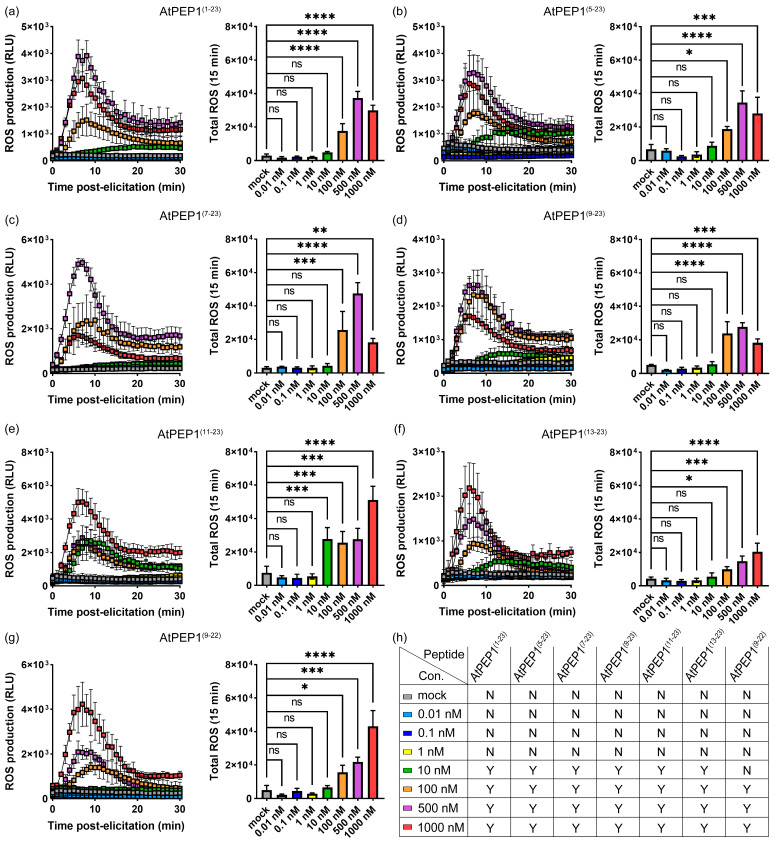
Dose-dependent ROS burst. Dynamic curves of ROS burst and total ROS within 15 min in Arabidopsis leaves induced by different doses of peptides AtPEP1^(1–23)^ (**a**), AtPEP1^(5–23)^ (**b**), AtPEP1^(7–23)^ (**c**), AtPEP1^(9–23)^ (**d**), AtPEP1^(11–23)^ (**e**), AtPEP1^(13–23)^ (**f**), and AtPEP1^(9–22)^ (**g**). Results are expressed as mean ± SD; “ns” indicates no significant difference compared to mock treatment. “*”, “**”, “***”, and “****” indicate significant differences compared to the control group at *p* < 0.05, *p* < 0.01, *p* < 0.001, and *p* < 0.0001 levels, respectively (one-way ANOVA, n = 4). (**h**) The occurrence of ROS burst. The symbol “N” represents no occurrence of ROS, while the symbol “Y” signifies the presence of ROS.

The peptide elicitor AtPEP1^(1–23)^ is capable of being recognized and bound by its receptors, leading to the generation of ROS burst at nanomolar or even picomolar concentrations [6,40,41,42]. Therefore, we conducted experiments to detect the ROS burst induced by AtPEP1^(1–23)^ and truncated peptides at various concentrations (Figure 9, Table S5). At a concentration of 10 nM, AtPEP1^(1–23)^ could trigger ROS burst, and as the concentration increased, the intensity of ROS burst also gradually increased, except for 1000 nM (Figure 9a). Although we observed an ROS burst curve with 10 nM AtPEP1^(1–23)^ treatment, there was no significant difference in total ROS within 15 min compared to the control group (Figure 9a). When the concentration of AtPEP1^(1–23)^ was greater than or equal to 100 nM, the total ROS within 15 min was significantly higher than the control (Figure 9a). Despite variations in maximum values observed, the patterns of ROS burst triggered by truncated peptides, namely, AtPEP1^(5–23)^, AtPEP1^(7–23)^, AtPEP1^(9–23)^, AtPEP1^(11–23)^, and AtPEP1^(13–23)^, were similar to that of AtPEP1^(1–23)^. These peptides were capable of triggering ROS bursts at a concentration of 10 nM (Figure 9a–f,h). However, AtPEP1^(9–22)^ induced ROS burst at 100 nM concentration, demonstrating comparatively lower sensitivity compared to the other peptides (Figure 9).

**Figure 10:**
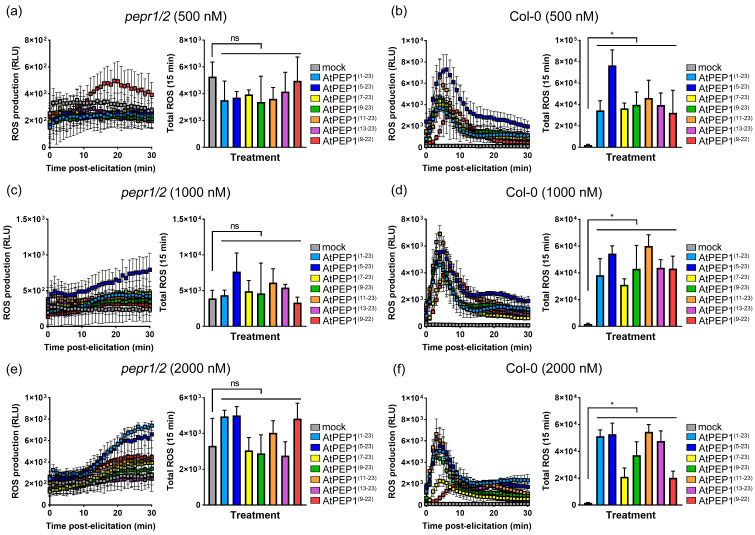
Dynamic curves of ROS burst and total ROS of *pepr1/2* mutant and wild-type Col-0. Panels (**a**,**c**,**e**) show the ROS results of *pepr1/2* mutant treated with peptides at concentrations of 500 nM, 1000 nM, and 2000 nM, respectively. Panels (**b**,**d**,**f**) show the ROS results of Col-0 treated with peptides at concentrations of 500 nM, 1000 nM, and 2000 nM, respectively. Total ROS represents the cumulative amount of active ROS generated within 15 min. Results are expressed as mean ± SD (n = 4); “ns” indicates no significant difference compared to mock; “*” indicates a significant difference to mock at *p* < 0.05 level.

Furthermore, we analyzed whether AtPEP1^(1–23)^ and truncated peptides rely on AtPEPR1 and AtPEPR2 to trigger ROS burst. None of the peptides used in this study were able to induce a significant ROS burst in the double mutant *pepr1/2*, even at concentrations as high as 2000 nM (Figure 10a,c,e, Table S6). However, they were able to trigger wild-type ROS burst (Figure 10b,d,f). These results suggest that the triggering of ROS burst by AtPEP1^(1–23)^ and truncated peptides is dependent on AtPEPR1 and AtPEPR2.

## 4. Discussion

Plants live in an environment with a diverse array of microorganisms, and have developed the capacity to timely detect potential infectious agents. Pattern-triggered immunity (PTI), as the first line of a plant’s defense system, is triggered by the recognition of both exogenous and endogenous elicitors. In plants, different elicitors are typically perceived by cell-surface pattern-recognition receptors (PRRs) [8,43,44,45], and may trigger convergent immune signaling events, including changes in cytoplasmic Ca^2+^ levels, activation of MAPK cascades, induction of defense-related genes, production of ROS burst and nitric oxide, deposition of callose to reinforce the cell wall, and stomatal closure to prevent pathogen entry [8,9,10,11]. 

The elicitor peptide AtPEP1^(1–23)^ can be recognized and bound by receptors AtPEPR1 and AtPEPR2, playing crucial roles in preventing pathogen invasion and root growth [6,40,41]. The *A. thaliana* genome contains eight PEP precursor genes (AtPROPEP1-8), and synthetic peptides AtPEP1-8 induce similar pattern-triggered immune responses [32]. The C-terminal of the synthesized AtPEP peptides possesses a conserved motif, SSG(x)_2_G(x)_2_N, while the N-terminal shows low conservation. We speculate that the low conserved N-terminal port of AtPEPs may be not important for their function. Experimental evidence demonstrates that AtPEP7, which is two amino acids shorter than the theoretically mature polypeptide at the N-terminal, still exhibits high activity [32]. This result proves the feasibility of our speculation.

The bacterial flagellin and elongation factor EF-Tu plays a vital role in the survival and pathogenicity of pathogens. By truncation, the highly conserved 22 amino acids (flg22) from flagellin and 18 amino acids (elf18) from EF-Tu, as core sequences, can trigger plant immune responses. The immune responses and mechanisms triggered by flg22 and elf18 have been extensively and deeply studied as PTI modes. To further elucidate the interaction mechanism between receptor and ligand, as well as the core sequence of the ligand, we conducted studies on two representative responses induced by AtPEP1^(1–23)^ through truncation, namely, root growth inhibition and ROS burst. 

Our previous research results indicate that AtPEP1^(1–23)^ significantly inhibits root growth by suppressing the expression of *DR5*, which leads to a reduction in auxin maximum leading to maturation of root tip cells [46]. We conducted external application of a series of truncated peptides and found that the truncation of the first eight amino acids at the N-terminus of AtPEP1^(1–23)^ does not affect its ability to inhibit the growth of primary roots (Figure 2a–d). However, further truncation resulted in a decrease in inhibitory efficiency (Figure 2e,f). In addition, truncation of the conserved amino acid at the C-terminus also reduces the inhibitory effect on the primary root (Figure 2g). Exogenous application of AtPEP1^(9–23)^ induces root hair production and inhibits the expression of *DR5* in root tip, similar to the results obtained with AtPEP1^(1–23)^ treatment (Figure 3 and Figure 5a). These results suggest that AtPEP1^(9–23)^ is the shortest sequence that maintains the inhibitory activity of peptide on the growth of primary roots.

In addition, AtPEP1^(1–23)^ regulates the flow of auxin and inhibits root growth by controlling the expression and activity of PIN2 and PIN3 [30]. Therefore, AtPEP1^(1–23)^ may not only regulate auxin synthesis in the roots but also its transport. PIN4, another PIN family member, mainly directs acropetal auxin flow from meristem to QC and promotes the formation of auxin gradient in roots [47,48,49]. According to our research findings, treatment with AtPEP1^(1–23)^, AtPEP1^(5–23)^, AtPEP1^(7–23)^, AtPEP1^(9–23)^, and AtPEP1^(11–23)^ leads to an increase in the expression of the *WOX5* gene in QC cells (Figure 5b). QC cells are a specialized group cells located at the root apex of plants that are responsible for maintaining and regulating the development and function of the root apical meristem. The *WOX5* gene plays a vital role in QC cells, participating in root meristem cell differentiation, growth, and maintaining the stability of the root system [35]. The increase in *WOX5* gene expression induced by peptide treatment may indicate the activation or transformation of QC cells into other cell types. According to the above research findings, it is speculated that AtPEP1^(1–23)^ and truncated peptides may affect auxin transport in the QC cells of plant root tips by regulating the PIN4 protein. However, further research is needed to elucidate the specific mechanisms of interaction between peptides, PIN4 protein, and auxin, as well as the regulatory mechanisms of these interactions on the expression of the *WOX5* gene.

In this study, we evaluated the impact of peptide treatment on early immune response by detecting ROS burst. The results show a close correlation between ROS burst and peptide concentration. AtPEP1^(1–23)^ and N-terminal truncated peptides cause ROS burst at a concentration of 10 nM, while the C-terminal truncated peptide, AtPEP1^(9–22)^, causes ROS burst at a concentration of 100 nM (Figure 9). These results suggest that truncating the N-terminal 12 amino acids of AtPEP1^(1–23)^ has little effect on its ability to trigger ROS burst, whereas even truncating a single amino acid from the C-terminal can affect its efficiency in triggering ROS burst. 

AtPEPR1 and AtPEPR2 with LRR extracellular domains are receptors for AtPEP1^(1–23)^ (Figure 7) [6,7]. In addition, the inhibitory effect on primary root growth and induction of ROS burst of truncated peptides also depends on AtPEPR1 and AtPEPR2 (Figure 6 and Figure 10). Truncation of more than eight residues significantly affects the ability of the peptides to inhibit primary root growth without affecting ROS burst (Figure 2 and Figure 9). These results indicate that peptides with N-terminal truncation of more than eight amino acids still retain biological activity and function. In leaves, both AtPEPR1 and AtPEPR2 function as receptors without obvious differences [6], while in roots, AtPEPR2 plays a dominant role [12,13,25,30]. We speculate that the differential dominance of AtPEPR1 and AtPEPR2 in different tissues is one of the reasons why AtPEP^(13–23)^ can induce ROS burst in leaves without inhibiting primary root growth. Additionally, the functional differences between truncated peptides may be attributed to their binding to receptor proteins or their activity. Therefore, this study conducted molecular docking analysis on three representative peptides AtPEP1^(1–23)^, AtPEP1^(9–23)^, and AtPEP1^(13–23)^ with receptors AtPEPR1 and AtPEPR2 to predict the binding modes of ligands and receptors. The results show that these three peptides are capable of binding to AtPEPR1 and AtPEPR2, with binding energies ranging from −232.05 to −203.73 kcal/mol (Figure 8). Specifically, both the residues Arg1 and Glu4 of AtPEP^(9–23)^ may establish hydrogen bonds with AtPEPR1 and AtPEPR2, respectively (Figure 8c). These hydrogen bonds play a crucial role in facilitating high-affinity binding between AtPEP^(9–23)^ and these receptors. The specificity of these interactions ensures the proper signal transduction and biological responses. Trimming off the amino acids between Arg1 and Glu4, namely, AtPEP^(11–23)^ and AtPEP^(13–23)^, reduces the inhibitory effects of these peptides on primary root growth (Figure 2). The C-terminal residue Asn15 of AtPEP^(9–23)^ may participate in hydrogen bonding interaction with Gln867 of AtPEPR1 (Figure 8c). Previous studies have shown that the extracellular domains of receptors are responsible for ligand recognition and binding [50,51]. According to the analysis results of TMHMM, it is known that the Gln867 residue is located in the intracellular kinase domain of AtPEPR1 (Figure 7). Therefore, the probability of interaction between Asn15 and Gln867 is low. However, Asn15 is crucial for the functionality of AtPEP^(9–23)^ (Figure 2 and Figure 9). It is speculated that Asn15 may affect peptide activity rather than interacting with the receptors. 

AtPEP1^(1–23)^ undergoes stepwise truncation, which alters its activity in triggering ROS burst and inhibiting primary root growth (Figure 2 and Figure 9). Therefore, the truncated peptide may exhibit changes in its activity, stability, and binding ability to receptors. Through truncating the peptide, the interaction mechanism between the receptor and ligand, as well as their regulation of cellular physiological processes such as cell proliferation and cell death, can be elucidated. The truncated peptides AtPEP1^(9–23)^, AtPEP1^(13–23)^, and AtPEP1^(9–22)^ used in this study may contribute to the analysis of the aforementioned questions. After truncation from AtPEP1^(1–23)^ to AtPEP1^(13–23)^, there is no significant change in the ability to trigger ROS burst, but the inhibitory effect on primary root growth is almost eliminated (Figure 2 and Figure 9). Immune responses triggered by AtPEP1^(1–23)^ play important roles in resistance against various pathogens [1,14,15,16,17]. Since both AtPEP1^(1–23)^ and AtPEP1^(13–23)^ rely on AtPEPR1 and AtPEPR2 to initiate immune response (Figure 10), the immune signaling pathway of the two peptides is the same. Compared to AtPEP1^(1–23)^, AtPEP1^(13–23)^ does not inhibit primary root and shoot growth (Figure 2 and Figure S1). Therefore, AtPEP1^(13–23)^ is more likely to be developed as a biopesticide. Furthermore, by rational design and modification of AtPEP1^(13–23)^ to increase its stability, activity, and binding characteristics with receptor, it can be better applied in practical production. Through in-depth research, the AtPEP1^(13–23)^ peptide is expected to become an environmentally friendly pesticide. In summary, our research findings have significant reference value in both theoretical research and practical applications.

## 5. Conclusions

Currently, AtPEP1^(1–23)^, containing 23 amino acids, is widely used. Through studying truncated peptide fragments, we have gained a better understanding of the contribution of each amino acid in the AtPEP1^(1–23)^ peptide to its function. It was found that truncating eight amino acids from the N-terminus, resulting in AtPEP1^(9–23)^, does not affect its ability to inhibit root growth and induce ROS burst, and can completely replace AtPEP1^(1–23)^. However, truncating 12 amino acids from the N-terminus, resulting in AtPEP1^(13–23)^, does not affect its ROS burst, but weakens its inhibitory effect on primary root growth. AtPEP1^(13–23)^ can be used as an immune elicitor to study immune responses without affecting plant growth. This study provides further insights for the regulation of plant root growth and adaptation to stress. The results of this study contribute to guiding the design and optimization of relevant peptide inhibitors and enhancers in future research and applications, aiming to achieve more effective regulation of plant root growth and adaptation to stress. Truncated peptide fragments may lead to decreased activity or affinity with receptors, ultimately leading to functional variations. However, the mechanisms of peptide–receptor interaction and how peptides regulate the inhibition of root growth by auxin still require further research.

## Data Availability

Data are contained within the article and Supplementary Materials.

## Supplementary Materials

The following supporting information can be downloaded at: https://www.mdpi.com/article/10.3390/antiox13050549/s1. Figure S1: The diameter of rosettes. Table S1: Statistical analysis for Figure 2. Table S2: Statistical analysis for Figure 4. Table S3: Statistical analysis for Figure 5. Table S4: Statistical analysis for Figure 6. Table S5: Statistical analysis for Figure 9. Table S6: Statistical analysis for Figure 10. Table S7: Statistical analysis for Figure S1.
