# Supplementary material for: The Truncated Peptide AtPEP1(9–23) Has the Same Function as AtPEP1(1–23) in Inhibiting Primary Root Growth and Triggering of ROS Burst
Source: Antioxidants (Basel). 2024 Apr 29;13(5):549. doi: 10.3390/antiox13050549 (PMC11117541; doi:10.3390/antiox13050549)
Supplement: Supplementary file 1 [file antioxidants-13-00549-s001.zip › Figure legend for Figure S1.pdf]

Figure S1. The diameter of rosettes. The 5-d-old wild-type plants were transferred onto half-strength MS agar medium with or without 500 nM peptide. Take photos of transferred seedlings daily and measure the diameter of rosettes on day 0 and day 4 after transferred using Image J. The experiment was repeated twice, with 20 seedlings transferred for each treatment in each repetition. "ns" indicated no significant difference, "*" and "****" indicated a significant difference at $p<0.05$ and $p<0.0001$ level, respectively.
