# Supplementary figures and images for: The Truncated Peptide AtPEP1(9–23) Has the Same Function as AtPEP1(1–23) in Inhibiting Primary Root Growth and Triggering of ROS Burst
Source: Antioxidants (Basel). 2024 Apr 29;13(5):549. doi: 10.3390/antiox13050549 (PMC11117541; doi:10.3390/antiox13050549)

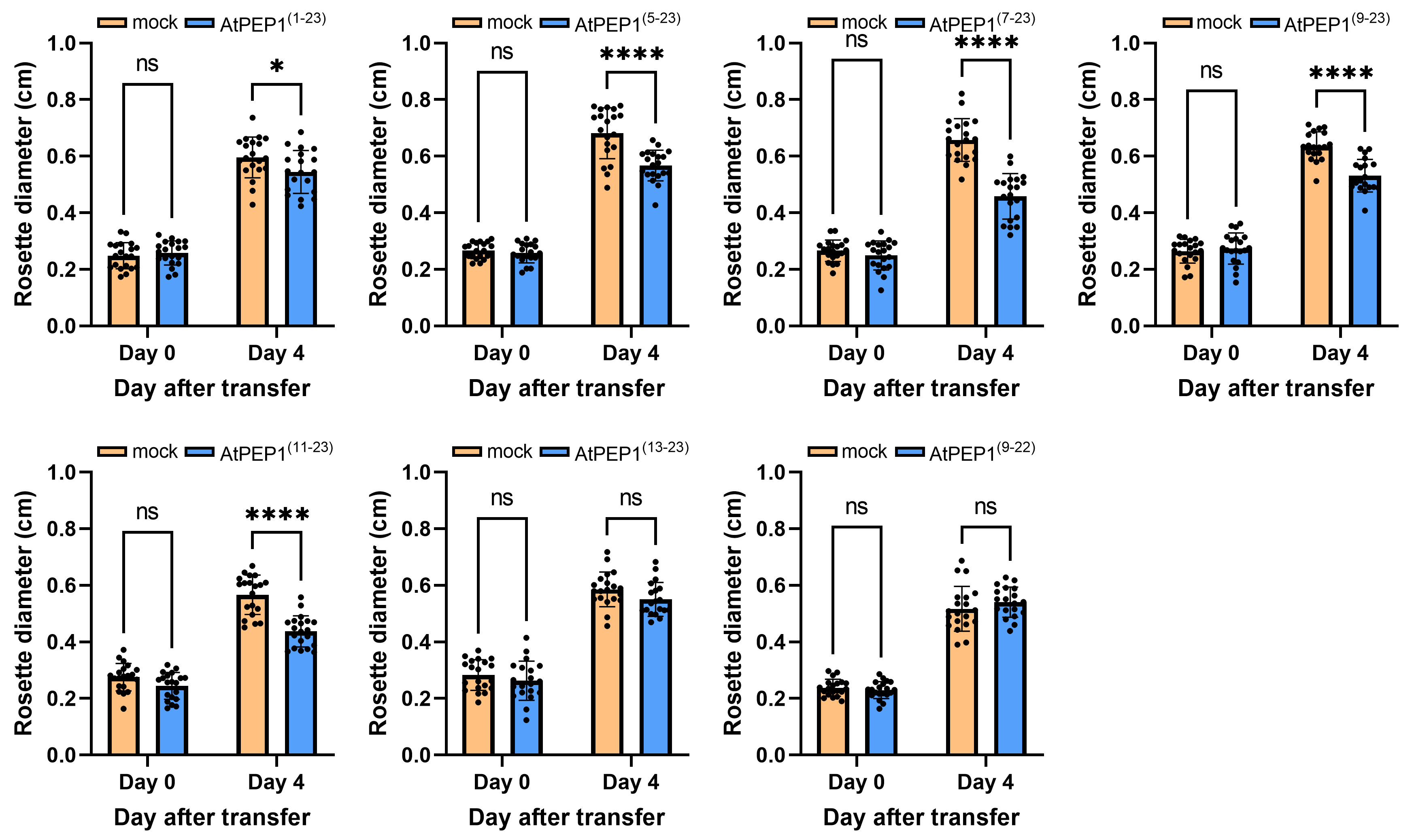

Supplement: Supplementary file 1 [file antioxidants-13-00549-s001.zip › Figure S1.tif]
